# How can the regulator show evidence of (no) risk selection in health insurance markets? Conceptual framework and empirical evidence

**DOI:** 10.1007/s10198-016-0764-7

**Published:** 2016-02-02

**Authors:** Wynand P. M. M. van de Ven, René C. J. A. van Vliet, Richard C. van Kleef

**Affiliations:** Department of Health Policy and Management, Erasmus University Rotterdam, Rotterdam, The Netherlands

**Keywords:** Health insurance, Risk equalization, Risk selection, I110, I130, I180

## Abstract

If consumers have a choice of health plan, risk selection is often a serious problem (e.g., as in Germany, Israel, the Netherlands, the United States of America, and Switzerland). Risk selection may threaten the quality of care for chronically ill people, and may reduce the affordability and efficiency of healthcare. Therefore, an important question is: how can the regulator show evidence of (no) risk selection? Although this seems easy, showing such evidence is not straightforward. The novelty of this paper is two-fold. First, we provide a conceptual framework for showing evidence of risk selection in competitive health insurance markets. It is not easy to disentangle risk selection and the insurers’ efficiency. We suggest two methods to measure risk selection that are not biased by the insurers’ efficiency. Because these measures underestimate the true risk selection, we also provide a list of signals of selection that can be measured and that, in particular in combination, can show evidence of risk selection. It is impossible to show the absence of risk selection. Second, we empirically measure risk selection among the switchers, taking into account the insurers’ efficiency. Based on 2-year administrative data on healthcare expenses and risk characteristics of nearly all individuals with basic health insurance in the Netherlands (*N* > 16 million) we find significant risk selection for most health insurers. This is the first publication of hard empirical evidence of risk selection in the Dutch health insurance market.

## Introduction

Since the early 1990s, consumers in an increasing number of countries have had a choice of health plan for basic health insurance. This is the case in, e.g., Belgium, Colombia, the Czech Republic, Germany, Israel, the Netherlands, Russia, Switzerland, and the United States of America (see, e.g., [20]). Consumer choice on the health insurance market is assumed to discipline insurers to increase efficiency and to be responsive to consumers’ preferences. Because unregulated competitive insurance markets result in risk-rated premiums and risk selection, all countries have regulated their health insurance markets to make basic health insurance affordable for all. To organize cross-subsidies, all of these countries implemented a risk equalization system in combination with regulations such as open enrollment (no refusal of eligible applicants), no exclusion of preexisting medical conditions, standardized insurance coverage, restrictions on the consumers’ out-of-pocket premiums, and restrictions on copayments. However, due to imperfections in the risk equalization systems in all countries there are groups of high-risk consumers (e.g., chronically ill people) who are substantially undercompensated, resulting in incentives for risk selection. Even in the presence of the above-mentioned regulations many forms of risk selection are possible (see “Forms of risk selection”). Risk selection may threaten the quality of care for chronically ill people, and may reduce the affordability and efficiency of healthcare. Therefore, an important question is: How can the regulator who is responsible for organizing the cross-subsidies show evidence of (no) risk selection in health insurance markets? Although it seems easy, showing such evidence is not straightforward.

The relevance of our paper is that, although we know that in many countries there are substantial *incentives* for risk selection, we do not know (1) whether or not risk selection really occurs in practice, and (2) if it occurs, to what extent, with which forms of selection, and with which negative effects for society. Because it is hard to observe risk selection, there may be ‘hidden negative effects’ of risk selection, as mentioned above. In this paper we provide a conceptual framework for showing evidence of risk selection that regulators can use. If regulators are able to show evidence of risk selection, they can take actions to reduce or avoid these negative effects. If regulators cannot show evidence of risk selection, the ‘hidden negative effects’ of selection may continue to exist.

The novelty of this paper is two-fold. First, we provide a conceptual framework for showing evidence of risk selection in competitive health insurance markets. Second, as an empirical illustration we apply one of the methods of measuring risk selection, using 2-year administrative data on healthcare expenses and risk characteristics of nearly all individuals with basic health insurance in the Netherlands (*N* > 16 million). The results are the first to show hard empirical evidence of risk selection in the Dutch health insurance market.

The structure of the paper is as follows. In the next section, we discuss risk equalization and several aspects of risk selection in competitive health insurance markets. Unlike most other studies, we give a definition of risk selection. For concepts, terminology, and definitions see Table 1. After that, we focus on showing evidence of risk selection, both conceptually and empirically. Finally, the conclusions are summarized, followed by a discussion and policy recommendations.

**Table 1:** Glossary

Cross-subsidies	We primarily focus on cross-subsidies from the low-risks to the high-risks. That is, we primarily focus on ‘risk-solidarity’ (and not on ‘income-solidarity’)
Equalization fund	The fund, managed by the regulator, out of which equalization payments are made. This fund can be filled with contributions by, e.g., consumers, insurers, government, and/or employers
Equalization payment	The payment per insured that an insurer receives from (if positive) or has to pay to (if negative) the equalization fund. In many countries the insurer may charge the insured an out-of-pocket premium
Health insurer	Risk-bearing entity that offers health plans, sometimes denoted as sickness fund
Health insurance agent	An agent or intermediary who advises consumers, and/or who sells health plans on behalf of the insurer; the agent may be authorized by the insurer to perform administrative functions. The insurer is the risk-bearing entity
Health plan	Health insurance product. All consumers who have the same ‘health plan’ have an identical contract with the insurer concerning benefits coverage, cost-sharing, quality, services, etc. An insurer may offer different health plans
Regulator	The entity that regulates and supervises the health insurance market, e.g., government, an entity empowered by government, or (a group of) employers (sometimes named sponsor, Health Alliance, Health Insurance Purchasing Cooperative, Connector, Health Insurance Exchange)
Residual expenses	The *actual* expenses minus the risk-adjusted *predicted* expenses
Risk adjuster	See 'risk adjustment'
Risk adjustment	A technique used to calculate risk-adjusted predicted health expenses based on the individual’s risk characteristics (‘risk adjusters’)
Risk equalization	A system of risk-adjusted equalization payments to and from insurers aimed at achieving the cross-subsidies from the low-risks to the high-risks as intended by the regulator
Risk selection	Actions (other than risk rating per health plan) by consumers and insurers with the goal and/or the effect that the cross-subsidies as intended by the regulator are not fully achieved

## Cross-subsidies and risk selection

To achieve the desired cross-subsidies the regulator requires that health insurers participate in a risk equalization system. Insurers with an overrepresentation of insured customers with high predicted expenses receive a higher risk-adjusted equalization payment from the equalization fund than insurers with an overrepresentation of low-risk insured. In addition, insurers may charge a premium to their enrollees. If the risk equalization is perfect, the cross-subsidies are achieved as intended by the regulator. Restrictions on the premium rates are then unnecessary. In practice, however, risk equalization is still imperfect and therefore most regulators aim to achieve the intended cross-subsidies by enforcing restrictions on the premium rates, such as a community rating or a premium bandwidth.

### Definition of risk selection

In Europe, the risk equalization ranges from primitive in Israel (age, gender, and region only) to quite sophisticated (with health indicators based on diagnostic information, prior utilization, and/or prior costs) in Belgium, Germany, and the Netherlands (see, e.g., [18, 22]). Although the research on risk adjustment started some 30 years ago, all risk equalization formulas currently used in practice substantially undercompensate selected groups of high-risk consumers, e.g., the chronically ill. For example, Table 2 presents the average under- and overcompensation per person in year t using the Dutch risk equalization formula-2014 for selected groups based on information from year* t* − 1.

**Table 2:** Average under- and overcompensation per person in year *t* for selected groups based on information from year *t* − 1, using the Dutch risk equalization formula-2014

Selected groups based on information from year* t* − 1	Estimated percentage of population	Undercompensation (−) in year* t*	Predictive ratio* in year* t*	Reduction of undercompensation compared with no risk equalization
Worst score physical health (SF-12)	18.9 %	−€670	0.85	–75 %
Contact with a medical specialist in the last 12 months	37.8 %	−€326	0.90	–75 %
Use of physiotherapy in the last 12 months	21.8 %	−€328	0.89	–71 %
At least one chronic condition	31.5 %	−€331	0.90	–80 %

Selected groups based on information from year* t* − 1	Estimated percentage of population	Overcompensation (+) in year *t*	Predictive ratio* in year *t*	Reduction of overcompensation compared with no risk equalization
No chronic condition	68.5 %	+€152	1.16	–66 %
Best score physical health (SF-12)	19.2 %	+€291	1.31	–71 %
No health care utilization in the last 12 months	19.5 %	+€298	1.51	–75 %
Highest education levels	22.8 %	+€142	1.10	–61 %

Because the Dutch government requires a community rating per health plan, for each selected group the average under- and overcompensation per person has been calculated as the average residual expenses per person. All presented under- and overcompensations are statistically significant at the 0.01 level, except for “highest education levels”, which are statistically significant at the 0.05 level

The Dutch risk equalization model of 2014 is based on the following risk characteristics, which over time have been added successively: age interacted with gender (1993), region (1995), source of income interacted with age (1999), pharmacy-based cost groups (2002), diagnoses-based cost groups (2004), socioeconomic status interacted with age (2008), multiple-year high costs (2012), yes/no student, and prior use of durable-medical-equipment (2014)

* The predictive ratio equals the ratio of the average *predicted* expenses and the average *actual* expenses of the individuals in the selected group

By enforcing restrictions on the premium rates, the regulator enforces the insurers to put over- and undercompensated groups of insured customers into one pool and to charge these heterogeneous risks the same premium. By doing so, the regulator aims at achieving *implicit* cross-subsidies as a complement to the *explicit* cross-subsidies that are realized via the risk equalization. However, these implicit cross-subsidies result in predictable losses and profits on selected groups of insured, which then provide the consumers and insurers with incentives to exploit that unpriced risk heterogeneity (i.e., the risk heterogeneity within each risk group as discerned in the risk equalization) and break these pooling arrangements [12]. For example, the over- and undercompensated insured may choose separate health plans that are attuned to their specific preferences. With community ratings per health plan the overcompensated insured then pay a low premium for their product, and the undercompensated insured pay a high premium. Despite the community rating requirement, the cross-subsidies as intended by the regulator are then not fully achieved. One can think of many actions that result in such market segmentation. Selection refers to these *actions* or to the *outcomes* of these actions [12]. We adjust Newhouse’s definition to the context of regulated competitive health insurance markets with risk equalization, and define risk selection as ‘actions (other than risk rating per health plan) by consumers and insurers with the goal and/or the effect that the cross-subsidies as intended by the regulator are not fully achieved’ [25]. Our definition of risk selection includes all forms of selection, such as adverse (risk) selection, preferred (risk) selection, direct selection, and indirect selection.

### Forms of risk selection

In Table 3, we discern four types of actions that in the case of *imperfect* risk equalization and *premium rate restrictions* can be qualified as risk selection. By requiring open enrollment and a mandate to buy health insurance, most regulators prohibit two straightforward forms of selection: (1) ‘insurers refusing high-risk applicants’ (type-1 action) and (2) ‘low-risk consumers refusing to buy health insurance’ (type-3 action). But many other forms of risk selection are not forbidden by most regulators. For example, an insurer offering the best care for chronically ill patients who are substantially undercompensated by the risk equalization may attract a disproportionally large number of undercompensated insured, and therefore has to increase his premium. Consequently, the cross-subsidies as intended by the regulator are not fully achieved. This example illustrates that it is not correct to associate risk selection exclusively with actions by *insurers* with the *goal* to attract overcompensated insured. Risk selection also comprises actions by *consumers* as well as the *effects* of actions (that were primarily not intended to selectively attract overcompensated insured).

**Table 3:** Four types of actions that in the case of *imperfect* risk equalization and *premium rate restrictions* can be qualified as risk selection

	Actions with the goal	Actions with the effect
Actions by insurers	Type-1 action Example: being non-responsive (e.g., via benefit design) to the preferences of unhealthy people with the *goal* to keep these people away from the health plan	Type-2 action Example: improving the quality of care for unhealthy people with the side-*effect* that the insurer attracts a disproportionally large number of these people
Actions by consumers	Type-3 action Example: healthy consumers choose a limited provider plan with a low premium with the *goal* to avoid paying a higher premium that contains (more) cross-subsidies to the unhealthy consumers (market segmentation)	Type-4 action Example: unhealthy consumers choose high-cost, high-quality plans more often than the healthy, with the *effect* that these groups end up in different pools with different premiums (market segmentation)

Healthy consumers are assumed to be overcompensated and unhealthy are assumed to be undercompensated

Although our definition of risk selection may seem quite broad, a more narrow definition would make no sense because often it is not possible to discern (1) whether an action is taken by the consumer or by the insurer, and whether (2) the non-achievement of the cross-subsidies as intended by the regulator is the goal or (only) the effect of an action. For example, the selection due to selective contracting can be the result of the action by an insurer (contracting only with a panel of selected providers) that was *not intended* to attract overcompensated insured, but it can also be the result of the actions of the overcompensated consumers choosing the health plan with a limited provider network. Often, it is a combination of actions by the insurer and the insured. It is also important to stress that the word ‘goal’ in the definition of risk selection does not imply that there is no risk selection if that goal is not achieved. There can be severe risk selection even if the ‘actions with the goal’ do not achieve their goal, e.g., because all insurers are equally successful in selection. In addition, there can be actions with the *effect* that the cross-subsidies as intended by the regulator are not fully achieved, while this was not the *goal* of these actions.

Note that the four types of actions in Table 3 can only be qualified as risk selection if the risk equalization is imperfect and if there are premium rate restrictions. In the case of *perfect* risk equalization, the cross-subsidies as intended by the regulator are fully achieved (no under- or overcompensations) and by definition, risk selection is nonexistent (except for actions by insurers who incorrectly think that the risk equalization is imperfect). In the case of *no* premium rate restrictions, insurers in a competitive market will adjust their premium rates to the under- and overcompensations by the risk equalization, rather than applying risk selection.^1^

Despite the open enrollment requirement there can be many different forms of risk selection in the case of imperfect risk equalization, for example, health plan discrimination (i.e., the offered health plans being attuned to the preferences of the different under- and overcompensated groups of insured), distorting the quality level of the offered plans, providing the doctors and hospitals with incentives for selection, selective advertising and marketing, and selection via insurance agents, group contracts, or supplementary insurances (see “Signals of risk selection”).

### Effects of risk selection

The effects of selection may be different for the different types of action, as discerned in Table 3. All forms of selection may result in market segmentation with the over- and undercompensated insured choosing different health plans with different (community-rated) premiums. Consequently, the cross-subsidies as intended by the regulator are then not fully achieved.

Another potential effect of selection, in particular of type-1 actions, is a reduction of efficiency. When the expected returns on selection, which are substantial (see, e.g., Shen and Ellis [17] and Table 2), exceed those on efficiency improvements, insurers are confronted, at least in the short term, with financial incentives to invest in selection rather than in improving efficiency. Even if all insurers are equally successful in this type of selection (and therefore no insurer has a selective risk composition of insured), their incentives for efficiency are reduced, at least in the short run.

The most worrisome form of selection is a specific form of type-1 action, i.e., service level distortion, e.g., by underprovision of services preferred by the undercompensated insured and overprovision of services preferred by the overcompensated insured (e.g., [2, 5, 7]. For this type of risk selection it is not necessary that insurers know *which* individuals are under- or overcompensated by the risk equalization. It is sufficient for them to know *that* patients with disease *X* who have relatively strong preferences for good quality of treatment *Y* are undercompensated. Insurers may then skimp the quality of care that is particularly used by the undercompensated, high-cost insured. They may also give poor service to the undercompensated insured and choose not to contract with providers who have the best reputations for treating their diseases. This, in turn, can discourage physicians and hospitals from acquiring such a reputation. That would be an undesirable outcome of a competitive healthcare system. Even if all insurers are equally successful in this type of selection, and therefore have the same risk composition of insured, this type of risk selection threatens the quality of care for the undercompensated patients.

Another possible outcome is that some insurers specialize in care for undercompensated high-risk patients and charge them a relatively high premium. In that case, the undercompensated high-risk patients receive good care and good services only if they are able and willing to pay the high premium.

In theory, type-1 actions, most of which are not in violation of the regulations, seem the most worrisome forms of risk selection. Nevertheless, in practice it is hard to disentangle the four types of selection actions. It is very hard to assess whether a health plan with a restricted panel of providers was set up with the goal to improve efficiency or to discourage undercompensated people from joining the health plan. Even more difficult is to show whether a potential improvement in healthcare has *not* been implemented because of efficiency (no cost-effective care), or because implementation would encourage undercompensated people to join the health plan. This illustrates that the effects of risk selection may be (largely) invisible: for example, we will never know how good the quality of care for the undercompensated high-cost patients would have been in the case of perfect risk equalization.

## Showing evidence of risk selection: A conceptual framework

Showing evidence of risk selection requires showing evidence of “actions with the goal and/or the effect that the cross-subsidies as intended by the regulator are not fully achieved”. Because it is hard to show evidence of the *goal* of certain actions, we will first concentrate on showing evidence of the *effects* of actions. In doing so, we first restrict ourselves to showing evidence that the cross-subsidies as intended by the regulator are not fully achieved. We discuss two methods of estimating risk selection (“Residual expenses” and “Overrepresentation of over- or undercompensated groups”). First, we discuss residual expenses as an estimate of risk selection (“Residual expenses”). Second, the level of overrepresentation of over- or undercompensated groups per insurer or health plan is discussed as an estimate of risk selection (“Overrepresentation of over- or undercompensated groups”). Third, we provide a list of signals of selection that can be measured and that, in particular in combination, can show evidence of risk selection (“Signals of risk selection”).

### Residual expenses

Showing evidence that “the cross-subsidies as intended by the regulator are not fully achieved” requires that it is known what these intended cross-subsidies are. These intended cross-subsidies^2^ can be derived from (1) the risk equalization payments per insured and (2) the restrictions on the premium rates. For example, in the case of community ratings per health plan an identical risk distribution across the health plans is implicitly assumed to yield the cross-subsidies as intended by the regulator. In most European countries the equalization payment per individual equals the risk-adjusted *predicted* expenses for that individual minus *p* % of the overall average expenses per person,^3^ with, e.g., *p* = 0 in Israel, *p* = 50 in the Netherlands, and *p* = 100 in Switzerland.^4^ In addition, the insurer may charge the insured a community-rated premium reflecting the insurer’s efficiency.^5^ This implies that in most European countries the cross-subsidies as intended by the regulator are such that the ‘residual expenses (i.e., actual expenses minus risk-adjusted predicted expenses) in the case of perfect risk equalization’ for each insured *in expectation* are zero, assuming average efficiency.

“Cross-subsidies such that the residual expenses on each insured *in expectation* are zero” imply that ex-ante the statistically *expected*/*predicted* residual expenses are zero for each insured. Because the unpredictable variation in individual residual expenses is large, ex-post there will always be a large variation in the actual residual expenses per *individual* insured, even with perfect risk equalization. Therefore, showing evidence of risk selection cannot be done on the basis of one individual insured who ex-post (by accident) has extreme positive or negative residual expenses. Showing evidence of risk selection requires a sufficiently large number of individuals and can only be done with a certain level of statistical significance.^6^

One could be inclined to measure risk selection by calculating for each insurer the average residual expenses of its insured.^7^ The intuitive idea to do so is that if an insurer has an overrepresentation of overcompensated insured, this insurer will have, after risk equalization, lower-than-average residual expenses per insured and vice versa. The conclusion could then be that, if these average residual expenses are different from zero for at least one insurer, with a certain level of statistical significance, there is risk selection because at least one insurer is over- or undercompensated and thus the cross-subsidies as intended by the regulator are not fully achieved.^8^ However, in most cases this conclusion is incorrect and the measure of risk selection is biased, as will be argued below.

#### Biased estimates of selection because of differences in insurers’ efficiency

Because the insurers’ average residual expenses are influenced by both selection and the insurers’ efficiency, these average residual expenses are biased estimates of risk selection if they are not adjusted for the differences in insurers’ efficiency. For example, negative average residual expenses per insurer can be the consequence of (1) being more efficient than average (and no risk selection), and/or (2) risk selection (and having average efficiency). Therefore, it is important to take care that the measure of risk selection is not biased by the insurer’s efficiency.^9^

Insurers’ efficiency has two components: (1) efficiency at the insurer level, i.e., the insurer provides healthcare efficiently or has selectively contracted efficient providers; and (2) efficiency at the insured level, i.e., the insured have a preference for efficiency.^10^ These two components can go together, but not necessarily. For example, an insurer with average efficiency but with an effective marketing campaign in creating a reputation of ‘delivering (or contracting) efficient and appropriate and no unnecessary care’ may attract many insured who prefer to make use of healthcare services in an efficient way and who avoid unnecessary care. Consequently, this insurer will have lower than average expenses within the risk groups used for the risk equalization. Although this insurer has a selective risk composition of insured, there is no risk selection because this situation could also occur in the case of perfect risk equalization,^11^ when the cross-subsidies as intended by the regulator are fully achieved.

#### Underestimation because positive and negative selection effects cancel out

Even if the average residual expenses per insurer would be adjusted for the differences in efficiency among insurers, they may underestimate the true risk selection. One reason is that several forms of risk selection, both positive and negative, may occur simultaneously; therefore, positive and negative selection effects may cancel out. For example, an insurer may have an overrepresentation of selected groups of *under*compensated insured (e.g., due to offering the best care for the chronically ill) as well as an overrepresentation of selected groups of *over*compensated insured (e.g., due to selective advertising).

#### Underestimation because of selection within the insurers’ portfolio

A second reason why the insurers’ average residual expenses may be an underestimation of the true risk selection is related to the level of measurement. The risk equalization is mostly done at the level of risk-bearing insurers, while each insurer is often allowed to offer several health plans with different premium rates. Because risk selection may (often) take place at the health plan level, ideally, the average residual expenses should be measured at the health plan level and not at the insurer level. However, often the regulator and researchers only have access to expenditures data at the insurer level. If an insurer has one health plan with positive risk selection and another health plan with negative risk selection, the positive and negative selection effects may (partly) cancel out at the insurer level. In that case, the average residual expenses at the insurer level underestimate the true risk selection. In reality there may be serious market segmentation *within* the insurer’s portfolio’, if the undercompensated insured choose a health plan with a high premium and the overcompensated insured choose a health plan with a low premium.^12^

#### Underestimation because selection actions may be ‘unsuccessful’

A third reason why the insurers’ average residual expenses may be an underestimation of the true risk selection is because “actions with the *goal*” may not be successful and therefore not reflected in the insurers’ average residual expenses. For example, in the extreme, if all insurers are equally successful in risk selection and have an identical risk composition of their insured, the average residual expenses are zero for each insurer, apart from differences in efficiency. Nevertheless, there may be substantial risk selection, with all of its negative effects (e.g., distorting the quality of care).

### Overrepresentation of over- or undercompensated groups

A second way of measuring risk selection is to measure whether insurers have, ideally per health plan, an overrepresentation of selected groups of insured who are over- or undercompensated (e.g., groups such as those illustrated in Table 2). However, this measure of risk selection is also an underestimation of the true risk selection because overcompensated subgroups may partly cancel out undercompensated subgroups, and because selection actions may be ‘unsuccessful’ (see “Underestimation because selection actions may be ‘unsuccessful’”).

In applying this measure of risk selection it is necessary to know which selected groups are over- or undercompensated by the relevant risk equalization formula (as illustrated in Table 2). A problem with applying this measure in practice is that often the necessary data are not routinely available. An option is to hold a health survey among enrollees of one or all insurers or health plans. However, such a survey may be quite costly and potentially subject to selection bias (due to selective response) and manipulation.

### Signals of risk selection

If the above-mentioned measures of risk selection indicate the existence of risk selection, this is sufficient for knowing that risk selection is indeed present. However, the reverse is not true. If the measures do not indicate that there is risk selection, this does not mean that risk selection is absent. Yet there still might be (substantial) selection (see “Residual expenses”). Therefore, as a third method we provide a list of signals of selection that can be measured and that, in particular in combination, can show evidence of risk selection.^13^ We do not pretend to present a complete list of all possible signals. We primarily focus on type-1 actions because these may have the most worrisome consequences (e.g., distorting the quality level of care). Measurement of these signals of risk selection is also useful in the case that the above-mentioned measures of risk selection indicate that risk selection is present, but it is still unknown which forms of risk selection this entails, and with which effects. It is important to note that the extent to which certain observed actions can (or cannot) be characterized as risk selection crucially depends on the quality of the risk equalization system. For example, actions with the effect of having only young insured can be considered as risk selection only if age is *not* included as a risk adjuster in the risk equalization formula. Because in most countries the model for calculating the risk equalization payment is continuously improved, the measurement of (signals of) risk selection is a dynamic process. Actions that are currently considered as risk selection may no longer be risk selection after improving the risk equalization. Therefore, for the right interpretation of observed actions, it is necessary to know which selected groups of insured are over- or undercompensated, and to what extent (see, e.g., Table 2).

#### Health plan differentiation via contracting and delivering care

In different countries, insurers have different tools for contracting, organizing, managing, and delivering healthcare.^14^ The use of each of these tools may result in health plan differentiation and market segmentation, as different (risk-)groups of insured prefer different health plans. Therefore, dependent on the quality of the risk equalization, the application of each of these tools can be considered as risk selection. For making a list of signals of such risk selection it is important to know (1) which selected groups of insured are over- or undercompensated, and (2) what degrees of freedom the insurers have in differentiating their health plans.

In several countries, insurers are free to negotiate with the providers of healthcare about the quality and price of healthcare, including the providers’ financial incentives (e.g., pay for performance, or risk sharing). Insurers and providers may agree on protocols for medical treatments and the level of efficiency of the care, i.e., the price-quality ratio, e.g., of implants, pharmaceuticals, medical devices, and diagnostic tests. Another degree of freedom is that insurers can selectively contract with preferred providers only, and can decide on the level of reimbursement in case an insured is treated by non-contracted providers. It is not difficult to imagine how these tools can be applied with the goal or the effect of market segmentation such that the over- and undercompensated people end up in different health plans with different premium rates.

The regulator can measure the following signals of risk selection. First, the regulator can monitor the quality of the contracted providers, the level of reimbursement of selected pharmaceuticals, the level of reimbursement of care received from selected non-contracted providers, and the rules for necessary pre-authorization.

A second option is that the regulator holds interviews with representative organized groups of undercompensated patients that negotiate with the insurers about price, quality, and the insurer’s purchasing strategy. The regulator could ask them questions such as: Which health plans do (not) allow you to have much influence on the quality of care, on the selected preferred providers, and on the composition of the supplementary insurances? Do you have the feeling of (not) being welcome with certain health plans?

A third option is that the regulator holds interviews with providers of care who specialize in treating chronic conditions of undercompensated patients. Questions could include: Is it difficult for you to get a contract with insurers? Which health plans in particular present a challenge? These answers can be compared with the answers by other providers.

Finally, the regulator can create opportunities for whistle-blowing, e.g., by employees of insurers who have ethical problems with their insurer’s policy and their own duties.

#### Health plan differentiation via service level

Health plan differentiation can also take place by differentiating the service levels of health plans, such as: having all contacts with the insured only via internet and email, rather than having an office building; the speed and quality of answering emails and phone calls; and advice to patients when the insurer acts as an intermediary for patients asking for guidance about the best providers or about waiting times.

Differentiation of service level can take place at a personal level. For example, based on administrative data such as costs and utilization from prior years, insurers may be able to qualify an insured as an over- or undercompensated risk type. If the insurer expects that a certain individual is overcompensated, the insurer may offer him/her short response times and excellent mediation when care is needed, and the opposite when the insurer expects that the individual is undercompensated.

The regulator can monitor these tools for risk selection by means of interviews with selected groups of insured or via ‘mystery’-insured: on the one hand, very healthy (overcompensated) persons, and on the other hand, unhealthy (undercompensated) patients with several chronic conditions.

#### Selective marketing, also by insurance agents

There are many ways that insurers can selectively market their health plans. In addition, many people do not buy their health plan directly from the insurer but via an insurance agent, i.e., a person or organization that advises and assists consumers regarding insurance products. Insurers often provide insurance agents with a bonus fee for each (new) applicant. Whereas insurers have to respect open enrollment, this generally does not apply to agents. Insurance agents can easily distinguish between over- and undercompensated individuals (e.g., just by observing and asking questions about health status) and use this information when channeling applicants to health plans.

The regulator can monitor this tool for risk selection by analyzing the marketing activities of all insurers and their insurance agents. In which media do they advertise? What is their marketing strategy? Who is the target group? What is the insurer’s image? Are over- and undercompensated people equally attracted by the marketing campaign? Do selected groups of consumers receive special (financial) benefits if they purchase a health plan, e.g., free supplementary insurance or rebates on other (insurance) products?

#### Selective enrollment and disenrollment

To measure signals of selective enrollment and disenrollment the regulator could submit so-called ‘mystery’-applications to insurers and insurance agents, and let ‘mystery’-insured ask for more information by letter, email, phone, and internet: this would seek to compare experiences from overcompensated (very healthy) persons and undercompensated persons (unhealthy patients with specific chronic conditions).

Another option is to hold interviews with insured consumers who switched insurers or health plans and ask them: Why did you switch? Were you not satisfied with the quality level of care that was delivered or contracted by your previous insurer or health plan? Did you have the feeling of not being welcome with your previous insurer? Did you have the feeling of being kicked out?

#### Supplementary insurances and other tie-in products

Supplementary insurances can also be an effective tool for risk selection. This holds true in particular if (1) health plans and supplementary insurances are (seemingly) sold as one product, and (2) no special regulation applies to supplementary insurances. The latter implies that insurers are free to require new applicants for supplementary insurances to fill out a health questionnaire, to reject applicants, and/or to charge risk-rated premiums for supplementary insurances. This is the case in, e.g., the Netherlands and Switzerland [23]. The outcomes of a health questionnaire for supplementary insurance may help insurers to distinguish between applicants who are expected to be over- and undercompensated for regulated basic health insurance. By rejecting high-risk individuals for supplementary insurances (or by charging them excessive premiums for supplementary insurances), an insurer will be unattractive for these individuals.

In addition, insurers may give special financial benefits to the overcompensated insured if they purchase a health plan, e.g., rebates on other insurance products such as car insurance, fire insurance, or travel insurance. As soon as these insured switch to another health insurer, they no longer receive the rebates on these other products. The Dutch government facilitated such market segmentation and risk selection by allowing that health plans may provide up to 10 % in rebates to members of a ‘group’. This stimulated the forming of selected risk-groups. About two-thirds of the Dutch population have purchased their health plan via a ‘group’. Such groups can be organized by any legal entity (e.g., employers, shops, sports clubs, patient organizations, and private initiatives). Whereas insurers have to respect open enrollment, groups are free to reject applicants. For example, anyone can organize a group of overcompensated individuals and negotiate with insurers about (financial) benefits for the group. There are many examples of risk selection in the Netherlands via groups [10].^15^

The regulator can monitor these forms of risk selection by closely monitoring the market and the insurers’ behavior with respect to supplementary insurances and other (insurance) products, ‘groups’, and via ‘mystery’-applications for supplementary insurances and health plans. Do only selected (overcompensated) groups of consumers receive special financial benefits if they purchase a health plan? Finally, in the case of strong signals of risk selection, the regulator can measure whether an insurer, a health plan, or a group has an overrepresentation of over- or undercompensated groups (see “Overrepresentation of over- or undercompensated groups”) and, if the regulator is authorized to do so, the regulator can ask for the reports of meetings of relevant employees working for the insurer.

#### Priorities

The measurement of all possible signals of risk selection is very costly. Therefore, the regulator should set priorities. The regulator should make a good estimate of the likelihood of different forms of risk selection and the seriousness of its consequences, both in the short and in the long run. For example, a form of risk selection that only results in one group of consumers paying 100 euro per year more than another group, could be considered lower than the social loss resulting from a form of risk selection that distorts the quality of care and thereby reduces or eliminates the access to good quality care for the underpriced high-risk patients. By multiplying the estimated probability of each form of risk selection with its estimated social loss, the regulator may give priority to potential signals of forms of risk selection with the highest expected social loss.

Effective supervision can also prevent undesired forms of risk selection. In any case, the regulator must have a permanent update of which selected groups of insured are over-and undercompensated by the current risk equalization, and to what extent.

### Showing the absence of risk selection is impossible

Showing the *absence* of risk selection requires showing the absence of “actions with the intention and/or the effect that the cross-subsidies as intended by the regulator are not fully achieved”.

If risk equalization is *perfect*, risk selection is absent. However, it is impossible to show that the risk equalization is perfect. Perfect risk equalization exists if and only if there exists no single group of over- or undercompensated insured. Because in principle the number of subgroups is unlimited, it is practically impossible to show that there exists no single group of over- or undercompensated insured.^16^

If risk equalization is *imperfect*, it is also impossible to show the absence of risk selection. In principle, the number of actions that can be qualified as risk selection is unlimited. It is impossible to show the absence of all these actions. Showing that all insurers or all health plans have an equal risk portfolio of insured is also no proof of the absence of risk selection, because all insurers or all health plans could be equally successful in risk selection. It could also mean that with one or more insurers there is both positive risk selection (e.g., an underrepresentation of chronically ill insured) and negative risk selection (e.g., an overrepresentation of low-educated low-income people), and that these selection effects cancel out. Finally, not rejecting the null-hypothesis “that a selected group of insured is not over- or undercompensated” with a certain level of statistical significance is not a proof that “the selected group of insured is not over- or undercompensated”. Possibly, this group is over- or undercompensated, but the size of the group is too small to come to statistically reliable conclusions, e.g., in the case of rare diseases.

The conclusion is that although the evidence of risk selection can be shown with a specified level of statistical significance, it is impossible to show the absence of risk selection.

## Showing evidence of risk selection among switchers: empirical evidence

In “Biased estimates of selection because of differences in insurers’ efficiency” we argued that, because of differences in insurers’ efficiency, the insurers’ average residual expenses are biased measures of risk selection. In this section we will present measures of risk selection that are not influenced by that bias. However, our estimates may underestimate the true level of risk selection for the reasons mentioned in the sections “Underestimation because positive and negative selection effects cancel out” through “Underestimation because selection actions may be ‘unsuccessful’”.

We used 2-year administrative data on healthcare expenses and risk characteristics of nearly all individuals with basic health insurance in the Netherlands (*N* > 16 million). For each individual we calculated the residual expenses in both 2008 and 2009 as the consumer’s *actual* health expenses minus his *predicted* expenses based on the risk equalization model of 2012. For each year, we know with which of the 25 insurers each consumer is enrolled.

Our estimates of risk selection are not affected by the “bias because of differences in insurers’ efficiency” for the following reasons. We can exclude any effect of efficiency at the *insured* level because at that time no insurer had a specific reputation of ‘appropriate and no unnecessary care’. In addition, we are not aware of any selection action that could have resulted in an overrepresentation of insured ‘who prefer to make use of healthcare services in an efficient way and have a low propensity to use healthcare services’. To eliminate the effect of efficiency at the *insurer* level, we restricted our analysis to the residual expenses of the 500,000 switchers on 1 January 2009 only. A switcher is defined as an insured who on 31 December 2008 is insured with another insurer than on 1 January 2009. For the group of consumers who newly enrolled in insurer *X,* we calculate the average residual expenses in the year *before* the switch (2008), and for the group of consumers who disenrolled from insurer *X* we calculate the average residual expenses in the year *after* the switch (2009). It is plausible to assume that these residual expenses, which are influenced by the average efficiency of all other insurers^17^ and not by the efficiency of insurer *X*, are all influenced by the national average efficiency. Then, we can interpret these average residual expenses of the switchers per insurer as an average over- or undercompensation by the risk equalization system. In the case of significant over- or undercompensation, by definition, there is risk selection.

Our results are presented in Table 4. For most health insurers we found significant risk selection among their switchers on 1 January 2009. In the year after disenrollment the overcompensation ranged from −192 to +129 euro per insured. Most remarkable is insurer 25, who had both the highest average overcompensation on new enrollees and the highest average undercompensation on those who disenrolled. This insurer also had the lowest ‘average residual expenses’ per insured for non-switchers.

**Table 4:** Average overcompensation (Euros) in 2008 of ‘new enrollees on 1 January 2009’ and average overcompensation in 2009 of ‘disenrollees on 1 January 2009’, per insurer, after applying the Dutch risk equalization model 2012 (excluding the costs of mental care)

Health insurer (in 2009)	Enrollees on 1 January 2009	Disenrollees on 1 January 2009
	Average overcompensation in the year ***before*** the switch (2008)	Average overcompensation in the year ***after*** the switch (2009)
1	123^*^	–27
2	35	–54
3	–45	–142
4	39^*^	17
5	77^*^	–5
6	68^*^	66^*^
7	45^*^	129^*^
8	60^*^	78^*^
9	132	–47
10	70^*^	–12
11	–10	–35
12	81^*^	41^*^
13	108^*^	5
14	75^*^	55^*^
15	112^*^	13
16	13	40
17	81^*^	38
18	123^*^	89^*^
19	197^*^	26
20	115^*^	58^*^
21	163^*^	–50
22	126^*^	57
23	116^*^	–3
24	76	30
25	201^*^	–192^*^

The insurers are ordered based on decreasing ‘average residual expenses’ in 2009 for the non-switchers (with insurer 25 having the lowest ‘average residual expenses’). The average expenses per insured in 2009 were 1570 euro

*Source* Van de et al. [24]

Negative overcompensation = undercompensation

* Significant (*p* < 0.05)

All estimates of risk selection as presented in Table 4 may underestimate the true risk selection for the three reasons mentioned in the sections “Underestimation because positive and negative selection effects cancel out” through “Underestimation because selection actions may be ‘unsuccessful’”. In addition, based on our results we do not know which forms of risk selection took place, and what their effects are. In other words, we now know that there is risk selection on the Dutch health insurance market, but we do not (yet) know to what extent it threatens the quality of care for chronically ill people, or reduces the affordability and efficiency of healthcare. To prevent any potential ‘hidden negative effects for society’ of selection from continuing to exist, the Dutch regulator may use the list of signals of selection that can be measured and that, in particular in combination, can show evidence of risk selection (see “Signals of risk selection”). In addition, the regulator can reduce the (potential) underestimation of the true risk selection in our estimates by repeating our analysis on the level of health plans rather than insurers. We did not have data at the level of the health plans at our disposal, but the regulator has the power to access these data. To test for stability, the regulator may also perform such analysis for consecutive years. Eventually, the regulator can take actions to reduce or avoid negative effects.^18^

## Conclusion and discussion

Risk selection is the Achilles heel of a competitive health insurance market with risk equalization and premium rate restrictions. Even with the best risk adjustment formulas currently in practice, the insured and insurers are confronted with substantial incentives for risk selection. If risk selection occurs, it may threaten the quality of care for chronically ill people, and may reduce the affordability and efficiency of healthcare. The two largest health insurers, as well as the National Association of Health Insurers in the Netherlands gave public warnings that insurers are financially discouraged to invest in good quality care for undercompensated patients.^19^ Therefore, an important question is: How can the regulator show evidence of (no) risk selection? Although this seems easy, the conclusion of our paper is that showing such evidence is not straightforward.

We provided a conceptual framework for showing evidence of risk selection in competitive health insurance markets. We defined risk selection as ‘actions (other than risk rating per plan) by consumers and insurers with the goal and/or the effect that the cross-subsidies as intended by the regulator are not fully achieved’.

A measure of risk selection could be the “average residual expenses per insured” calculated for each insurer. Because an insurer’s residual expenses are influenced by both selection and the insurer’s efficiency, this measure is a biased indicator of selection. We suggested two methods to measure risk selection that are not biased by the insurers’ efficiency (see “Residual expenses” and “Overrepresentation of over- or undercompensated groups”). However, these measures underestimate the true risk selection. Therefore, we also provided a list of signals of selection that can be measured and that, in particular in combination, can show evidence of risk selection (see “Signals of risk selection”). It is impossible to show the absence of risk selection (see “Showing the absence of risk selection is impossible”).

Because in most countries the model for calculating the risk equalization payment is continuously improved, the measurement of (signals of) risk selection is a dynamic process. Actions that today are qualified as risk selection may no longer be risk selection after improving risk equalization.

Finally, we empirically estimated risk selection among switchers, taking into account the insurers’ efficiency. Based on 2-year administrative data on healthcare expenses and risk characteristics of nearly all individuals with a basic health insurance in the Netherlands (*N* > 16 million), we find significant risk selection for most health insurers. The estimated risk selection may underestimate the true risk selection. After 25 years of discussion about risk selection, this is the first hard empirical evidence of risk selection in the Dutch health insurance market.

Based on our results we now know that there is risk selection in the Dutch health insurance market, but we do not (yet) know to what extent it threatens the quality of care for chronically ill people, or reduces the affordability and efficiency of healthcare. To prevent potential ‘hidden negative effects for society’ of selection from continuing to exist, the Dutch regulator may use the list of signals of selection that can be measured and that, in particular in combination, can show evidence of risk selection (see “Signals of risk selection”). In addition, the regulator can reduce the (potential) underestimation of the true risk selection in our estimates by repeating our analysis on the level of health plans rather than insurers. To test for stability, the regulator may also perform such analysis for consecutive years.^20^ Eventually, the regulator can take actions to reduce or avoid negative effects.

Because the Netherlands has an advanced risk-equalization formula, it is likely that risk selection also occurs in other countries with similar regulation, such as Germany, Israel, Switzerland, and the United States (Medicare Advantage; Health Insurance Exchanges). Regulators in these countries should be eager to counteract risk selection and its negative effects.

## Policy recommendations

Based on our study we come to the following policy recommendations. First, because measuring all signals of risk selection is very costly, the regulator should set priorities. The regulator should make a good estimate of the likelihood of different forms of risk selection and the seriousness of the consequences, both in the short and long run. Second, the regulator should permanently monitor the market, in particular, as long as insurers do not advertise for all groups of chronically ill people, e.g. “Come with us because we have contracted the best doctors for your disease”. Third, a necessary condition for monitoring risk selection is that the regulator always has a permanent update of the extent to which selected groups of insured are over- or undercompensated by the current risk equalization (such as is illustrated in Table 2). Fourth, the measurement of risk selection should ideally be done at the health plan level rather than the insurer level. Finally, because most forms of risk selection in most countries are not a violation of the legislation, it is not straightforward that the regulator has sufficient tools to counteract undesired risk selection. Therefore, it is recommended to make an inventory of the tools that the regulator has available to counteract undesired forms of risk selection, and if necessary, to extend these tools or to take other measures. It goes without saying that the best way to prevent risk selection is by reducing or eliminating the *incentives* for risk selection, ideally by improving the risk equalization.

## Highlights

- With imperfect risk equalization there are incentives for risk selection.
- It is not easy to disentangle risk selection and the insurers’ efficiency.
- Many signals of risk selection can be measured.
- It is impossible to show the absence of risk selection.
- Our empirical results provide evidence of risk selection among switchers.

## Footnotes

1 There may be some risk selection if the costs of further premium differentiation are too high.

2 Additional subsidies, such as income-related subsidies to low-income people, are not relevant for our discussion of risk selection.

3 In the USA's Medicare Advantage system, the risk equalization payment equals a certain percentage of the predicted expenses in traditional Medicare.

4 In the Netherlands, *p* = 0 for children up to the age of 18; in Switzerland, children up to the age of 18 are exempted from risk equalization.

5 In Israel, insurers are not allowed to charge a premium. Dutch insurers are not allowed to charge a premium to children. In Switzerland, there are different community-rated premiums for the age groups 0–18, 19–25, and 26 + per insurer per canton. In the health insurance exchanges in the USA, the premium may be conditioned on age (1:3), smoking (1:1.5), family size, and geography, but not on other risk characteristics.

6 Nevertheless, one person can be sufficient for the *existence* of risk selection, e.g., an insurer’s action with the goal that an insured who is expected to be undercompensated will disenroll.

7 The insurers’ average residual expenses of their insured could be interpreted as (a proxy for) the financial result they would achieve under identical premiums and identical administrative costs (including additional revenues, e.g., due to interest).

8 The average residual expenses for *selected* groups of insured in the population (e.g., those with the worst health status) indicate that, with a certain level of statistical significance, there are *incentives* for risk selection (because some *selected* groups are over- or undercompensated). For an example, see Table 2.

9 The importance of disentangling the effects of selection and efficiency is nicely illustrated by McGuire et al. [7] as follows: “Ellis and McGuire [4] measure predictability, predictiveness, and the consequent incentives to ration services among plans competing in Medicare using data from traditional Medicare (not the managed care component for which data were not available). Cao and McGuire [2] in Medicare and Eggleston and Bir [3] in employer-based insurance find patterns of spending on various services consistent with service-level selection among competing at-risk plans. Ellis et al. [5] rank services according to incentives to undersupply them. Consistent with service-level selection, they show that HMO-type plans tend to underspend on services (in relation to the average) just as the selection index predicts. This pattern of spending is not observed among enrollees in unmanaged plans. An alternative interpretation, however, is that HMO plans are better at managing diseases that tend to be predictable, i.e., chronic illnesses where the ability to manage care is more feasible, and so reduce spending more for these diseases than for others in relation to less-managed plans. This latter interpretation is supported by the findings of Newhouse et al. [14].”

10 The insured may differ in tastes or in their preference for efficiency. For example, some insured may (ceteris paribus) have a low propensity to use healthcare services or prefer to make use of healthcare services in an efficient way, while other consumers may have opposite preferences.

11 We implicitly assume that (1) the regulator does not want to give lower equalization payments for insured who ceteris paribus prefer to make use of health care services in an efficient way; and that (2) the medical expenses function is additively separable in a set of risk factors for which the regulator wants to compensate the insurers via the risk equalization and a set of risk factors for which this is not the case (such as the ‘efficiency at the insured level’). If the second assumption is not fulfilled, it is impossible to get a precise estimate of risk selection because efficiency and risk selection cannot be disentangled [16].

12 A successful example of this type of risk selection is the building of so-called ‘conglomerates of insurers’ by Swiss insurers. In 2007, the most successfully risk-selecting conglomerate in Switzerland had 14 different insurers with very different premiums ranging from very cheap to rather expensive, and the salesmen of this conglomerate were very successful in ‘channeling enrollees to the insurer with a premium that best corresponded to their expected expenses’ [22]. Despite the community rating per insurer, the cross-subsidies as intended by the regulator are then not achieved.

13 Studies providing empirical evidence of signals of risk selection are e.g., Shmueli and Nissan-Engelcin [19], Mehrotra et al. [8], Bauhoff [1], Grunow and Nuscheler [6], Riley et al. [15], McWilliams et al. [9], and Newhouse et al. [13]. For a list of anecdotal evidence of risk selection, see e.g., Van de Ven et al. [21, 22].

14 For a list of such tools, see e.g., Van Kleef et al. [25] and Van de Ven et al. [23].

15 This type of selection could be easily avoided by prohibiting such types of rebates. However, this would reduce the consumers’ option to form powerful consumer groups that can effectively negotiate with the insurers.

16 This also implies that it is impossible to show the absence of *incentives* for risk selection.

17 To verify that the new enrollees and those who disenrolled were not disproportionally distributed among the other insurers, we performed two calculations. We calculated for each insurer the weighted average of the financial results (2008-data) of the 24 insurers where its new enrollees came from. This measure ranged among the 25 insurers from −9 to +6 euro (with one exception: −29 euro) and was never statistically significant. Similarly, the weighted averages of the financial results (2009-data) of the 24 insurers to which the disenrollees switched ranged from −15 to +16 euro and were never statistically significant.

18 For potential actions to do so, see e.g., Van de Ven and Ellis [20].

19 Achmea (2011), Letter of Achmea to the members of Parliament, 7 September 2011 (obtainable from the Clerk of Parliament or from the first author); and speech by Ab Klink, member of the Board of VGZ at the 14th Clingendael European Health Forum, The Hague, 26 March 2014, see https://www.youtube.com/watch?v=EPeXKppJeUE, and the letter of Zorgverzekeraars Nederland sent to the Minister of Health, 18 July 2014 (see ZN-Journaal nr. 30–31, www.zn.nl).

20 After finishing our study (in 2014), the Dutch regulator performed a similar analysis as we did for several consecutive years and found similar results (as in Table 4), which were quite stable over time. The regulator also announced plans to do the same analysis at the level of health plans (rather than insurers) and also to use a selection of the ‘list of signals of selection’ as mentioned in this paper. See Nederlandse Zorgautoriteit [11].

## References

[1] Bauhoff S (2012). Do health plans risk-select? An audit study on Germany's Social Health Insurance. J. Pub. Econ..

[2] Cao Z, McGuire TG (2003). Service-level selection by HMOs in Medicare. J. Health Econ..

[3] Eggleston K, Bir A (2009). Measuring selection incentives in managed care: Evidence from the Massachusetts state employees insurance program. J. Risk Insur..

[4] Ellis RP, McGuire TG (2007). Predictability and predictiveness in health care spending. J. Health Econ..

[5] Ellis RP, Jiang S, Kuo TC (2013). Does service-level spending show evidence of selection across health plan types?. Appl. Econ..

[6] Grunow M, Nuscheler R (2014). Public and private health insurance in Germany: The ignored risk selection problem. Health Econ..

[7] McGuire TG, Newhouse JP, Normand SL, Shi J, Zuvekas S (2014). Assessing incentives for service-level selection in private health insurance exchanges. J. Health Econ..

[8] Mehrotra A, Grier S, Dudley RA (2006). The relationship between health plan advertising and market incentives: Evidence of risk selective behavior. Health Aff..

[9] McWilliams JM, Hsu J, Newhouse JP (2012). New risk-adjustment system was associated with reduced favorable selection in Medicare Advantage. Health Aff..

[10] Nederlandse Zorgautoriteit: Verdiepend onderzoek, Naleving acceptatieplicht door zorgverzekeraars. Nederlandse Zorgautoriteit, Utrecht (2014)

[11] Nederlandse Zorgautoriteit: Kwantitatief onderzoek naar risicoselectie. http://www.nza.nl/publicaties/1048188/Rapport_kwantitatief_onderzoek_naar_risicoselectie (2015). Accessed 1 July 2015

[12] Newhouse JP (1996). Reimbursing health plans and health providers: Efficiency in production versus selection. J. Econ. Lit..

[13] Newhouse JP, Price M, Huang J, McWillimas JM, Hsu J (2012). Steps to reduce favorable selection in Medicare Advantage largely succeeded, boding well for health insurance exchanges. Health Aff..

[14] Newhouse, J.P., McWilliams, J.M., Price, M., Huang, J., Fireman, B., Hsu, J.: Do Medicare advantage plans select enrollees in higher margin clinical categories? J. Health Econ. **32**(6) 1278–1288 (2013) 10.1016/j.jhealeco.2013.09.003 PMC3855666 24308879

[15] Riley GF, Levy JM, Montgomery MA (2009). Adverse selection in the Medicare prescription drug program. Health Aff..

[16] Schokkaert E, Dhaene G, van de Voorde C (1998). Risk adjustment and the trade-off between efficiency and risk selection: An application of the theory of fair compensation. Health Econ..

[17] Shen Y, Ellis RP (2002). How profitable is risk selection? A comparison of four risk adjustment models. Health Econ..

[18] Shmueli A (2015). On the calculation of the Israeli risk adjustment rates. Eur. J. Health Econ..

[19] Shmueli A, Nissan-Engelcin E (2013). Local availability of physicians’ services as a tool for implicit risk selection. Soc. Sci. Med..

[20] Van de Ven, W.P.M.M., Ellis, R.P. Adjustment in competitive health insurance markets. In: Culyer, A.J., Newhouse, J.P. (eds.) Handbook of Health Economics (Chapter 14), pp. 755–845. Elsevier, Amsterdam (2000).

[21] Van de Ven, W.P.M.M., Beck, K., Buchner, F., Chernichovsky, D., Gardiol, L., Holly, A., Lamers, L.M., Schokkaert, E, Shmueli, A., Spycher, S., Van de Voorde, C., van Vliet, R.C.J.A., Wasem, J., Zmora, I.: Risk adjustments and risk selection on the sickness fund insurance market in five European countries. Health Policy **65**, 75–98 (2003) 10.1016/s0168-8510(02)00118-5 12818747

[22] Van de Ven WPMM, Beck K, Van de Voorde C, Wasem J, Zmora I (2007). Risk adjustment and risk selection in Europe: Six years later. Health Policy.

[23] Van de Ven WPMM, Beck K, Buchner F, Schokkaert E, Schut FT, Shmueli A, Wasem J (2013). Preconditions for efficiency and affordability in competitive healthcare markets: Are they fulfilled in Belgium, Germany, Israel, The Netherlands and Switzerland?. Health Policy.

[24] Van de Ven WPMM, Van Kleef RC, Van Vliet RCJA (2013). Risicoselectie bij overstap zorgverzekeraar. Econo. Stat. Ber..

[25] Van Kleef RC, Van Vliet RCJA, Van de Ven WPMM (2013). Risk selection in a regulated health insurance market: A review of the concept, possibilities and effects. Expert. Rev. Pharmacoecon. Outcomes Res..

